# Central Retinal Artery Occlusion: Current Practice, Awareness and Prehospital Delays in Switzerland

**DOI:** 10.3389/fneur.2022.888456

**Published:** 2022-05-23

**Authors:** Elena Ardila Jurado, Veit Sturm, Florian Brugger, Krassen Nedeltchev, Marcel Arnold, Leo H. Bonati, Emmanuel Carrera, Patrik Michel, Carlo W. Cereda, Manuel Bolognese, Sylvan Albert, Friedrich Medlin, Christian Berger, Ludwig Schelosky, Susanne Renaud, Julien Niederhauser, Christophe Bonvin, Marie-Luise Mono, Biljana Rodic, Alexander A. Tarnutzer, Guido Schwegler, Stephan Salmen, Andreas R. Luft, Nils Peters, Jochen Vehoff, Georg Kägi, The Swiss Stroke Registry Investigators

**Affiliations:** ^1^Department of Neurology and Stroke Center, Cantonal Hospital St. Gallen, St. Gallen, Switzerland; ^2^Department of Ophthalmology, Cantonal Hospital St. Gallen, St. Gallen, Switzerland; ^3^Department of Neurology, Kantonsspital Aarau, Aarau, Switzerland; ^4^Department of Neurology, Inselspital, Bern University Hospital, University of Bern, Bern, Switzerland; ^5^Department of Neurology and Stroke Center, University Hospital Basel and University of Basel, Basel, Switzerland; ^6^Department of Neurology, Hôpitaux Universitaires de Genève, Geneva, Switzerland; ^7^Stroke Center, Neurology Service, Lausanne University Hospital, Lausanne, Switzerland; ^8^Stroke Center, Neurocenter of Southern Switzerland, Lugano, Switzerland; ^9^Neurocenter, Cantonal Hospital of Lucerne, Lucerne, Switzerland; ^10^Cantonal Hospital Graubuenden, Chur, Switzerland; ^11^Stroke Unit, Cantonal Hospital Fribourg, Fribourg, Switzerland; ^12^Spital Sarganserland Grabs, Grabs, Switzerland; ^13^Division of Neurology, Cantonal Hospital Münsterlingen, Münsterlingen, Switzerland; ^14^Division of Neurology, Cantonal Hospital Neuchatel, Neuchâtel, Switzerland; ^15^Stroke Unit, Groupement Hospitalier de l'Ouest Lémanique, Nyon, Switzerland; ^16^Hôpital du Valais, Sion, Switzerland; ^17^Stadtspital Waid und Triemli, Zurich, Switzerland; ^18^Cantonal Hospital Winterthur, Winterthur, Switzerland; ^19^Cantonal Hospital of Baden, Baden, Switzerland; ^20^Division of Neurology, Hospital Limmattal, Schlieren, Switzerland; ^21^Spitalzentrum Biel, Biel, Switzerland; ^22^Department of Neurology and Clinical Neuroscience Center, University Hospital Zurich and University of Zurich, Zurich, Switzerland; ^23^Cereneo Center for Neurology and Rehabilitaiton, Vitznau, Switzerland; ^24^Department of Neurology, Hirslanden Clinic, Zurich, Switzerland

**Keywords:** central retinal artery occlusion (CRAO), incidence, ischemic stroke, awareness, prehospital

## Abstract

**Background and Purpose:**

Central retinal artery occlusion (CRAO) often leads to permanent monocular blindness. Hence, early recognition and rapid re-perfusion is of paramount importance. This study aims to describe prehospital pathways in CRAO compared to stroke and study the knowledge about CRAO.

**Methods:**

(1) Description of baseline characteristics, prehospital pathways/delays, and acute treatment (thrombolysis/thrombectomy vs. standard of care) of patients with CRAO and ischemic stroke registered in the Swiss Stroke Registry. (2) Online survey about CRAO knowledge amongst population, general practitioners (GPs) and ophthalmologists in Eastern Switzerland.

**Results:**

Three hundred and ninety seven CRAO and 32,816 ischemic stroke cases were registered from 2014 until 2019 in 20 Stroke Centers/Units in Switzerland. In CRAO, 25.6% arrived at the hospital within 4 h of symptom onset and had a lower rate of emergency referrals. Hence, the symptom-to-door time was significantly longer in CRAO compared to stroke (852 min. vs. 300 min). The thrombolysis/thrombectomy rate was 13.2% in CRAO and 30.9% in stroke. 28.6% of the surveyed population recognized CRAO-symptoms, 55.4% of which would present directly to the emergency department in contrast to 90.0% with stroke symptoms. Almost 100% of the ophthalmologist and general practitioners recognized CRAO as a medical emergency and 1/3 of them considered IV thrombolysis a potentially beneficial therapy.

**Conclusions:**

CRAO awareness of the general population and physician awareness about the treatment options as well as the non-standardized prehospital organization, seems to be the main reason for the prehospital delays and impedes treating CRAO patients. Educational efforts should be undertaken to improve awareness about CRAO.

## Introduction

The central retinal artery is a branch of the ophthalmic artery supplying the inner retina, including the macula and fovea. Thromboembolic occlusion of the central retinal artery (CRAO) is a special form of stroke with an estimated incidence of 1.8/100,000, reaching up to 10/100,000 in the age group over 80 years (1–3). Clinically, CRAO is characterized by sudden and painless unilateral loss of vision, often described as a “curtain coming down” or a generalized darkening. Spontaneous recovery of vision occurs in <30%, and persistent monocular visual loss has a significant impact on the quality of life (4–6).

The duration of retinal ischemia is the most important determinant of visual outcome. Irreversible damage of the retinal ganglion cells occurs within 12 to 15 min of non-perfusion (7). Similar to ischemic stroke, time to irreversible damage largely depends on the completeness of occlusion and collaterals. In rhesus monkeys, irreversible ischemic damage of the retina after CRAO occurs within 4 h (8), highlighting the possibility of a therapeutic window. However, therapeutical approaches aiming to improve retinal perfusion (i.e., carbogen, acetazolamide, topical beta-blockers, ocular massage, and anterior chamber paracentesis) lack efficacy so far (9–11). This also holds true for fibrinolytic therapies in CRAO, where randomized controlled evidence of an effect on visual outcome is still missing (9, 12–14). One eminent methodological issue of all studies on thrombolysis in CRAO was a broad time window for inclusion, reaching far beyond 4 h from symptom onset. These broad inclusion criteria regarding symptom onset to treatment time may be partly explained by non-standardized diagnostic and therapeutic concepts, leading to important prehospital delays. At this time point, it is largely unknown how many patients with CRAO are being referred to and finally thrombolysed at stroke centers/stroke units (SCs/SUs) in Switzerland. However, in Switzerland a thrombolysis can only be conducted in SCs/SUs, therefore all CRAO patients who receive a thrombolysis are registered in the Swiss Stroke Register (SSR).

Therefore, we aim to describe (i) the current practice and number of patients with CRAO treated in SCs/SUs in Switzerland, (ii) the prehospital delays, and iii) prehospital pathways compared to patients with ischemic stroke based on the data from the SSR. In the second part of this study, we aim to assess the level of awareness of CRAO symptoms and handling of these patients amongst the general population, general practitioners (GPs) and ophthalmologists.

## Materials and Methods

### Patients With CRAO and Ischemic Stroke From the Swiss Stroke Registry (SSR)

In the first part of the study, we analyzed registered cases with CRAO and ischemic stroke between January 1. 2014 and Dezember 31. 2019. We describe and compare the prehospital pathways including emergency services as well as non-emergency service pathways. Patients with CRAO were categorized into two groups: those receiving thrombolysis and those with standard of care treatment. Because recovery of visual acuity after CRAO is not collected in a standardized way in the SSR, outcome data are not provided.

The SSR is the only national database that prospectively collects data from acute stroke patients admitted within 7 days from symptom onset to SCs/SUs in Switzerland. These SCs/SUs are certified according to national Swiss Stroke Unit and Stroke Center criteria, and are in line with those of the European Stroke Organisation (15). Patients were informed of the collection of their data in the SSR. Patients who did not consent to use of their data for research purposes (1,483 patients with ischemic stroke and 13 patients with CRAO) and patients who were diagnosed from a different disease after the stroke work-up were excluded. The study was approved by the SSR steering committee in cooperation with the ethics committee of eastern Switzerland (EKOS) and was performed in accordance with the ethical standards as laid down in the 1964 Declaration of Helsinki and its later amendments. CRAO patients were orally informed about the off-label use of thrombolysis.

### Level of CRAO Awareness Amongst the General Population-, General Practitioners and Ophthalmologists

In the second part of the study, we assessed awareness about symptoms and treatments of CRAO amongst the general population, GPs and ophthalmologists. The survey was carried out between March 2019 and July 2020. The population-level was assessed with an electronic questionnaire, which was completed by patients and their companions in the waiting area of an interdisciplinary outpatient clinic [neurosurgery, rheumatology, neurology (not including stroke outpatient clinic)] in the cantonal hospital of St. Gallen. The questionnaire was completed without assistance.

The population-based questionnaire consisted of 16 questions concerning CRAO symptoms, warning signs, and approach in case of permanent and temporary acute unilateral visual loss as well as approach in case of stroke symptoms.

In order to assess the level of awareness about CRAO amongst GPs and ophthalmologists, a link to the questionnaire was sent by e-mail to the GPs and ophthalmologists in the catchment area of St. Gallen to be filled out online. The questionnaire for the GPs and ophthalmologists consisted of 16 and 11 questions, respectively, regarding symptoms of CRAO, differential diagnosis, etiology, approach in case of persistent and temporary acute visual loss, therapy options, and time windows. The complete questionnaire can be found in the Supplementary Material (see Online Resource 1).

The data that support the findings of this study are available from the corresponding author upon reasonable request.

### Statistical Analysis

#### Part 1

Baseline characteristics are summerized descriptively using frequencies and percent except for age, which is presented as mean and standard deviation (SD). Prehospital data are presented as frequencies and percent except symptom onset to door, door to needle, and door to groin puncture time which are presented as median with interquartile range (IQR). In order to compare the baseline characteristics from (1) CRAO patients with and without revascularisation and (2) from CRAO patients with ischemic stroke patients, the Chi-Square test (if the contingency tables contained more than two columns or more than two rows and at least one cell assignment was smaller than five, the Fisher's exact test was chosen) for binary and the two-sided student's *t*-test for continuous variables were used. *P*-values of < 0.05 were considered significant.

Since this study was not designed as a confirmatory study, but to identify patterns, it was not necessary to make an adjustment for multiple testing. The significance tests used therefore have a descriptive character.

Patients with missing data were included in the analysis. The missing data does not exceed the threshold of 30%, except for data of transportation. The missing data were excluded from the analysis.

#### Part 2

The binary variables were expressed in frequencies and percentages. The answers of the population were analyzed with a binary logistic regression with the answers (yes/no) as dependent and age as a continuous independent variables. A *p*-value < 0.05 was considered significant.

Analysis of the SSR data and the survey were performed with IBM SPSS statistical software version 25 (IBM Co. Armonk, NY, USA).

## Results

### Part 1: Patients With CRAO and Ischemic Stroke Patients From the Swiss Stroke Registry

#### Baseline Characteristics

In the SSR, 397 (37.5% female) patients with CRAO and 32,816 (42.5% female) patients with ischemic stroke were registered during the time period reviewed here. The mean age was 71.8 (13.7) and 72.4 (13.7) years, respectively. Baseline characteristics are comparable between CRAO patients receiving the standard of care or acute revascularisation, except that standard of care CRAO patients were treated significantly more often with direct oral anticoagulants (DOAC) (*p* = 0.006).

Female sex, atrial fibrillation and diabetes mellitus, were more common in patients with ischemic stroke compared to patients with CRAO. Hyperlipidemia, prosthetic heart valves, peripheral artery disease, antithrombotic treatment, treatment with DOAC and lipid-lowering treatment were more common in patients with CRAO (Table 1).

**Table 1:** Baseline characteristics from CRAO and ischemic stroke patients.

	**CRAO (all)**	**CRAO (revascularised)**	**CRAO (standard of care)**	**CRAO revasc. vs. standard of care *p*-value**	**Stroke**	**CRAO (all) vs. stroke *p*-value**
Total number (*n*)	397	43	282		32,816	
Mean age yr (SD)	71.8 (13.7)	71.6 (9.5)	71.7 (14.3)	0.948	72.4 (13.7)	0.429
Female sex—*n* (%)	148 (37.3)	14 (32.6)	106 (37.6)	0.748	13,959 (42.5)	0.036*
**Medical history—*****n*** **(%)**						
- Any Stroke	60 (15.8)	7 (16.3)	45 (16.6)	0.957	5,598 (18.4)	0.193
- TIA	30 (7.9)	4 (9.3)	20 (7.4)	0.756	1,938 (6.4)	0.228
- Intracranial hemmorrhage	3 (0.8)	1 (2.3)	2 (0.7)	0.359	558 (1.8)	0.132
- Coronary heart disease	84 (22.1)	8 (18.6)	64 (23.6)	0.468	5,768 (18.9)	0.119
- Atrial fibrillation	51 (13.4)	3 (7.0)	41 (15.1)	0.234	7,503 (24.6)	0.000*
- Prosthetic heart valve	23 (6.0)	0	16 (5.9)	0.142	919 (3.0)	0.001*
- Low output (LVEF)	4 (1.3)	0	4 (1.7)	1.000	721 (2.8)	0.123
- Hypertension	272 (71.6)	29 (67.4)	200 (73.8)	0.383	22,782 (74.7)	0.169
- Hyperlipidemia	273 (71.8)	33 (76.7)	194 (71.6)	0.483	19,913 (65.4)	0.009*
- Diabetes mellitus	55 (14.5)	3 (7.0)	43 (15.9)	0.124	6,537 (21.4)	0.001*
- Current smoking	86 (22.6)	7 (16.3)	68 (25.1)	0.208	6,444 (21.3)	0.526
- Peripheral artery disease	34 (9.0)	2 (4.7)	29 (10.8)	0.279	1,720 (5.7)	0.006*
**Medication at presentation—*****n*** **(%)**						
- Antithrombotic	157 (40.6)	23 (53.5)	108 (38,8)	0.069	11,415 (34.8)	0.018*
- Vitamin K antagonist	31 (7.9)	1 (2.3)	25 (8.9)	0.225	2,012 (6.4)	0.228
- DOAC	37 (12.0)	0	32 (15.5)	0.006*	2,531 (7.7)	0.005*
- Antihypertensive	238 (61.0)	25 (58.1)	176 (62.9)	0.552	18,917 (60.6)	0.879
- Antilipids	149 (38.2)	17 (39.5)	102 (36.4)	0.694	9,737 (31.2)	0.003*

* *p < 0.05. CRAO, central retinal artery occlusion; DOAC, direct oral anticoagulants; TIA, transient ischemic attack*.

#### Prehospital Pathways

One hundred and fifty nine (41.0%) of the patients with CRAO were referred from another hospital, 116 (29.9%) were self-referrals, this includes all patients who presented to the emergency room without referral from a general practitioner, another specialist or hospital. Eighty one (20.9%) were referred from the GP, and only 23 (5.9%) from emergency services. For CRAO-patients receiving thrombolysis, emergency referrals doubled [5 (11.6%)] at the expense of referrals from the GP [6 (14.0%)]. Most of the CRAO patients [185 (83.0%)] used private transportation (self, taxi, relatives), and only 37 (16.6%) came by emergency services. Comparing thrombolysed versus standard of care patients, private transportation was still the most commonly used 17 (70.8%), with a trend to more transportation by emergency services 7 (29.2%).

Median symptom onset to door time in patients with CRAO was 788 min (168 min in thrombolysed and 918 min in standard of care patients). Only 86 (26.0%) of patients arrived at the hospital within 4 h after symptom onset.

Prehospital pathways of patients with CRAO compared to patients with ischemic stroke shows a lower rate of emergency referrals (5.9% vs. 41.5%) and higher rates of referrals from GPs (20.9% vs. 12.4%), from other hospitals (41% vs. 18.3 %) and self-referrals (30.3% vs. 21.3%). Transportation was more often individual or by taxi (83.0% vs. 30.8%) and less often by ambulance (16.6% vs. 65.3%). The symptom-to-door time was much longer in patients with CRAO compared to strokes (788 min vs. 265 min) (Table 2).

**Table 2:** Prehospital pathway from patients with CRAO and ischemic stroke.

	**CRAO (all)**	**CRAO (revascularised)**	**CRAO (standard of care)**	**CRAO revasc. vs. standard of care *p*-value**	**Ischemic stroke**	**CRAO (all) vs. stroke *p*-value**
**Referral—*****n*** **(%)**						
- Emergency service	23 (5.9)	5 (11.6)	14 (5.0)	0.153	13,104 (41.5)	0.000*
- General practitioner	81 (20.9)	6 (14.0)	57 (20.4)	0.319	3,917 (12.4)	0.000*
- Self referral	116 (29.9)	13 (30.2)	95 (34.1)	0.622	6,726 (21.3)	0.000*
- Other stroke unit or center	3 (0.8)	0	3 (1.1)	1.000	819 (2.6)	0.024*
- Other hospital	159 (41.0)	17 (39.5)	107 (38.4)	0.882	5,772 (18.3)	0.000*
- In-hospital event	6 (1.5)	2 (4.7)	3 (1.1)	0.134	1,252 (4.0)	0.015*
**Transport—*n*°** **(%)**						
- Ambulance	37 (16.6)	7 (29.2)	26 (15.4)	0.127	16,256 (65.3)	0.000*
- Helicopter	1 (0.4)	0	1 (0.6)	0,0093	967 (3.9)	0.008*
- Other (taxi, self, relatives…)	185 (83.0)	17 (70.8)	142 (84)	0.142	7,680 (30.8)	0.000*
Wake-up CRAO / unknown symptom onset—*n* (%)	96 (28.6)	1 (2.5)	83 (31.1)	0.000*	12,043 (37.4)	0.001
Time from symptom onset to door—median (IQR)	788 [235, 1,553]	168 [117, 206]	918 [349, 1,832]	0.000	265 [89, 900]	0.000*

* *p < 0.05. CRAO, central retinal artery occlusion; IVT, intravenous thrombolysis; IAT, intra-arterial thrombolysis; IQR, interquartile range*.

#### Initial Assessment, Acute and Post-acute Treatment and Follow-Up Imaging of Patients With CRAO and Ischemic Stroke

CT- or MR-angiography showed a stenosis of at least 50% in 89 (30.7%), and an occlusion in the corresponding territory in 24 (8.3%) CRAO patients. The rate of carotid stenosis observed in CRAO patients is significantly higher than in stroke patients. In 32.7% of the CRAO patients, a concomitant (silent) brain ischemia was detected. The thrombolysis rate was 13.2% (43) for CRAO patients (8.9% systemic- and 4.3% intra-arterial thrombolysis), which is significantly lower than the observed thrombolysis rate in stroke patients (30.9%). Among CRAO patients with a symptom-to-door time of <4 h, the thrombolysis rate was 45% (36.0). Of the remaining patients who arrived beyond 4 h after symptom onset, only 4 patients (1.7%) were thrombolysed. The median time from symptom onset to systemic thrombolysis was 225 min. with a range from 135 min. to 793 min. The median door to needle time in CRAO was 9 min shorter than in stroke patients (40 min.). The median time from symptom onset to intra-arterial thrombolysis (groin puncture time) in CRAO patients was 298 min. with a range from 195 to 445 min (Table 3). Thrombolysis rate among patients arriving within 4 h increased from 33% (2/6) in 2014 to 50% (12/24) in 2018 with a slight decrease to 38.5% (10/26) in 2019.

**Table 3:** Initial Assessment, acute and post-acute treatment and follow up imaging in patients with CRAO and ischemic stroke.

	**CRAO (all)**	**CRAO (revascularised)**	**CRAO (standard of care)**	**CRAO revasc. vs. standard of care *p*-value**	**Ischemic stroke**	**CRAO (all) vs. stroke *p*-value**
**First imaging—*n*°** **(%)**						
- CT	262 (86.4)	36 (87.8)	218 (85.8)	0.734	22,593 (78.8)	0.001*
- MRI	41 (13.6)	5 (12.2)	36 (14.2)	0.734	6,063 (21.2)	0.001*
**First imaging result—*n*°** **(%)**						
- Stenosis in suspected territory	89 (30.7)	10 (23.8)	79 (32.6)	0.255	3,999 (15.4)	0.000*
- Occlusion in suspected territory	24 (8.3)	5 (11.9)	19 (7.9)	0.371	9,409 (36.2)	0.000*
**Follow-up imaging result—*n*°** **(%)**						
- No acute lesion	101 (60.1)	26 (68.4)	73 (58.9)	0.291	2,744 (12.9)	0.000*
- Ischemia	55 (32.7)	10 (26.3)	41 (33.1)	0.433	15,714 (74.0)	0.000*
- Ischemia with hemorrhagic transformation	2 (1.2)	1 (2.6)	1 (0.8)	0.415	2,342 (11.0)	0.000*
- Intracranial hemorrhage	1 (0.6)	1 (2.6)	0	0.235	221 (1.0)	1.000
Systemic thrombolysis—*n*° (%)	29 (8.9)				6,454 (20.6)	0.000*
Time from symptom onset to IVT—median (IQR)	225 [187, 260]				140 [103, 210]	0.09
Time from door to IVT—median (IQR)	31 [23, 69]				40 [29, 60]	0.683
Intraarterial thrombolysis—*n*° (%)	14 (4.3)				4,857 (15.5)	0.000*
Time from symptom onset to IAT—median (IQR)	298 [235, 335]				256 [180, 465]	0.307
Time from door to IAT—median (IQR)	169 [68, 306]				91 [63, 127]	0.171
Carotid endarterectomy—*n*° (%)	36 (9.8)	2 (4.8)	30 (11.5)	0.279	982 (3.4)	0.000*
Carotid stenting—*n*° (%)	17 (4.7)	1 (2.4)	13 (5.1)	0.701	453 (1.6)	0.000*
pfo closure—*n*° (%)	0				250 (0.9)	0.083

* *p < 0.05*.

#### Etiology of CRAO and Ischemic Stroke

The most common etiology of CRAO was large artery atherosclerosis in 115 (30.5%) patients, followed by cardioembolism in 40 (10.6%) patients. The etiology in ischaemic stroke differs slightly from CRAO. The most prevalent etiology in ischaemic stroke was cardioembolism with a frequency of 27.4% (8,287), followed by large artery atherosclerosis with 14.9% (4,500) (see Online Resource 2.1).

### Part 2: Survey

#### Population

Three hundred and fifty subjects (42.3% male) with a mean age of 44 [16–85] years filled in the questionnaire. Two participants who did not complete at least 50% of the questions were excluded from the analysis.

One hundred of 350 subjects (28.6%) recognized an acute, unilateral loss of vision as a symptom of ischemia of the eye. 136 (39.5%) have already heard about retinal infarction. Half of the surveyed population [167 (47.7 %)] would seek the GP's or ophthalmologist's advice. Two hundred and thirty nine (69.7%) identified a transient loss of vision as potentially harmful and would consult a doctor, and the majority thought that treatment of an acute visual loss should be done in the first 2 h after symptom onset 205 (60.8%).

Younger people were more knowledgeable regarding symptoms of CRAO and action to be taken compared to older people (Table 4).

**Table 4:** Awareness of the population dependent on age.

**Questions**	**Overall positive answers (%)**	**OR (95% CI)**	***p***
Sudden unilateral loss of vision as symptom	100 (28.6)	1.028 (1.011–1.044)	0.001*
CRAO is considered as a medical emergency	221 (63.1)	1.019 (1.005–1.034)	0.009*
I would go immediately to the next emergencies	194 (55.4)	1.019 (1.005–1.033)	0.008*
Make an appointment with my optician/GP	167 (47.7)	1.009 (0.995–1.023)	0.193
**I have heard before about retinal infarction from:**			
- Periodicals	50 (14.3)	0.974 (0.955–0994)	0.009*
- Television	47 (13.4)	0.99 (0.971–1.01)	0.340
- Internet	40 (11.4)	1.023 (1.0–1.046)	0.05
- Friends, relatives	67 (19.1)	0.992 (0.975–1.009)	0.346
- GP	19 (5.4)	1.0 (0.971–1.03)	0.999
I would go immediately to the next emergencies in case of stroke	304 (88.9)	0.985 (0.964–1.007)	0.109

* *p < 0.05.* *CRAO, central retinal artery occlusion; GP, general practitioner; p < 0.05. OR > 1: positive association between younger age and correct answer*.

In order to compare the behavior of the population in case of a stroke, compared to CRAO, we asked the surveyed subjects what would be the next step in case of stroke symptoms. Three hundred and four (89.9%) of the subjects would present to the emergency room in case of stroke, compared to 194 (55.4%) in case of symptoms related to CRAO.

#### Ophthalmologist

Seventy ophthalmologists working in private practice were contacted by email, of whom 49 responded and 38 completed at least half of the questionnaire. From the 65 hospital-based ophthalmologist contacted by e-mail, 30 responded to the questionnaire and 29 could be included in this study.

Sixty six of 67 (98.5%) surveyed ophthalmologists recognize CRAO as a medical emergency, and 43 (64.2%) of them would transfer their patients to the emergency room of a stroke center. Fifty one (76.1%) of them consider the symptoms to be reversible under therapy in the first 4 h after symptom onset.

Thirty-two (47.8%) of the ophthalmologists consider the early treatment with aspirin as potentially effective. Fifty-four (80.6%) of them consider conservative therapies (reducing intraocular pressure, eye massage, etc.) as an appropriate treatment in case of CRAO. However, only 16 (23.9%) would consider systemic thrombolysis and 28 (41.8%) intra-arterial thrombolysis (see Online Resource 3.2).

#### General Practitioners

The questionnaire was sent by e-mail to 520 GPs in Eastern Switzerland. We received 109 answers. After excluding participants who did not answer at least half of the questionnaire, we could include 102 general practitioners. We observed a good knowledge of the differential diagnosis in case of visual loss. All of them recognize CRAO as an emergency, and the majority (88.2%) recognized a sudden unilateral loss of vision as the typical symptom in CRAO.

CRAO can be considered as an infrequent disease seen by GPs. Only 30 (29.4%) claim to have personal experience with patients with CRAO, with 58 (56.9%) of them treating less than one patient per year. The most common approach was to transfer the patient to the next stroke center, with 61 (59.8%) positive answers. The majority of the GPs consider intra-arterial thrombolysis as potentially effective, with 55 (53.9%) positive answers. Fifty-two (51.0%) GPs consider an early treatment with aspirin as a correct treatment, and 38 (37.3%) of them would take systemic thrombolysis into consideration (see Online Resource 3.3).

## Discussion

Our study in patients with CRAO registered in the SSR provides important insights on the number of patients treated in SCs/SUs, the number of thrombolysed patients, prehospital and in-hospital pathways, and delays.

With an estimated incidence of 1.8–1.9/100'000 and based on the census from the swiss population with 8,606,033 inhabitans in 2019, ~156 patients with CRAO per year can be expected in Switzerland, but only half of them are registered in the SSR. The incidence of the registered cases in the SSR starts with 0.29 with 24 cases registered in the SSR in 2014 and reaches 1.51, with 130 cases in 2019. This may have two main reasons: First, the SSR was launched in Switzerland in 2014 with continuous improvement of completeness of data over the past years. Second, due to the growing evidence that patients with CRAO might benefit from thrombolysis, their number might have increased in SUs/SCs. Nevertheless, it is very likely that there is a significant number of patients with CRAO who are not being seen in a SC/SU. As thrombolysis can only be performed in SCs/SUs in Switzerland, all patients with CRAO who received a thrombolysis are registered in the SSR and represented in this study.

In the SSR cohort, large artery atherosclerosis was the most common cause of CRAO, which is in accordance with the literature where carotid artery stenosis in the context of CRAO is found in up to 40% of cases (2, 14, 16–23). Non of the revascularised CRAO patients was under DOAC treatment because of the contraindication.

Importantly, concomitant (silent) brain ischemia was detected in 32% of the patients, which fits well to the reported numbers in the literature (23–25). This is relevant for two reasons: (i) brain imaging is mandatory before considering thrombolysis, and (ii) speedy aetiological stroke work-up of patients with CRAO is important to prevent further vascular events.

For patients with CRAO who are referred to a SC/SU, the delay from symptom onset to door is remarkably longer compared to patients with ischemic stroke (788 min. vs. 265 min.). Factors for this delay in patients with CRAO might be the low rate of referrals by the emergency services and the high number of self-referrals (30%), and secondary transports (83%). Self-referred patients had a median delay from symptom onset to door of 618 min., which suggests insufficient knowledge about the condition at the population level. On the other hand, in-hospital delays in SCs/SUs do not seem to represent a relevant problem because the median door to needle time was 9 min faster than in stroke patients.

The prehospital delays seems to be the main reason for withholding thrombolysis from patients with CRAO. There is a remarkable difference in the thrombolysis rate in patients arriving within 4 h after symptom onset, with 45% receiving thrombolysis when compared to patients arriving beyond this time window, where only 1.7% of them received thrombolysis.

The efficacy of thrombolysis in CRAO lacks evidence from randomized clinical trials so far. However, data from non-randomized studies and meta-analyses strongly suggest that thrombolysis of CRAO within 4.5 h from symptom onset improves visual outcome (9, 12, 26). A recent article shows a recovery rate of nearly 0.8 in patients thrombolysed within 90 min of symptom onset, which drops down to 0.2 if thrombolysis is performed beyond 4 h 30 min (26). A recovery rate of 0.2 after 4.5 h corresponds to the natural history of CRAO. Therefore, improving prehospital organization and knowledge about CRAO being a special form of stroke seems crucial. This has also been highlighted in a recent scientific statement from the American Heart Association (27).

The first important step in the prehospital cascade is to recognize CRAO symptoms and the knowledge of acute CRAO management in order to reduce prehospital delays. Earlier studies emphasized the importance of awareness of diseases like TIA or ischemic Stroke among GPs (28, 29). A low awareness about the disease as well as right approach lead to treatment delays (28, 29). Even though thrombolysis for CRAO is not approved yet, nearly half of the patients who arrive within 4 h after symptom onset are thrombolysed in Switzerland with a steady increase over the past years. Hence, decreasing prehospital delays in CRAO patients would increase thrombolysis rate and allow inclusion in randomized controlled trials. Furthermore, early stroke work-up and initiation of secondary preventive treatments will reduce the risk of further ischamic events.

In our survey on healthy subjects, we have seen that the knowledge about the unilateral loss of vision in the context of CRAO is rather poor, and therefore only about half of the surveyed population would seek immediate care in an emergency department in case of an acute unilateral visual loss. This is in contrast to the awareness of stroke symptoms where 88.9% of the surveyed population would seek for treatment in an emergency department.

Overall, the knowledge of symptoms of CRAO among the population is low with better awareness in younger people. Another possible delay comes from the GPs where the knowledge of CRAO symptoms and therapy options is good, but the short time window for therapeutical options is less well-appreciated. This adds well to the findings from the registry that many patients arrive at the hospital far beyond the possible window of opportunity for thrombolysis. The most common observed approach in case of CRAO under the ophthalmologist is also the transfer of the patients to the next stroke center; the aceptance of thrombolysis as a possible treatment option is however low. We acknowledge that thrombolysis is not an approved therapeutical option in CRAO yet. Nevertheless, the evidence for a beneficial effect of thrombolysis is growing, and the percentage of concomitant silent strokes is remarkably high in these patients, which underpins the urge for acute neurological assessment and treatment. Furthermore, good randomized controlled trials with CRAO-thrombolysis need a speedy prehospital organization to be able to restrict inclusion to a favorable time window of 4.5 h from symptom onset. The high thrombolysis rate in CRAO patients, who reach a SC/SU within 4 h from symptom onset unterlines the acceptance of thrombolysis as a possible treatment option in current daily practice. Ophthalmological and neurological assessment, in order to exclude possible differential diagnosis, as well as immediate brain and vascular imaging, should be standardized, similar to the stroke pathways in these patients. Losing time in this situation is losing one eye with all its consequences on quality of life (30).

Many educational efforts have been made and are still undertaken to promote awareness about other diseases like TIA, stroke, and heart attack. Information campaigns about stroke should also focus on the topic of sudden unilateral visual loss. Reducing prehospital delays will potentially increase therapeutic options and, with this, increase the chance of visual recovery.

### Limitations

The SSR data is collected prospectively, however the data were analyzed retrospectively which leads to some limitations. The SSR doesn't include outcome measures for vision. Therefore, this study is not able to answer this question. The survey among the population was conducted in the waiting room of our outpatient clinic. Although stroke patients were not included, this fact as well as the overrepresentation of younger people may have lead to a selection bias with an overestimation of the knowledge about CRAO.

## Conclusion

Although CRAO is a serious condition affecting multiple aspects of daily living and the quality of life (30), specific prehospital pathways to improve symptom to door time are not defined. Prehospital delays occur at multiple levels (e.g., population, treating physicians). Hence, CRAO should be part of the prehospital concepts of ischemic stroke. Although the effect of thrombolysis has not be proven so far in large clinical trials, meta-analysis describe a benefit in the first hours after symptom onset in CRAO patients. This is reflected in the current practice in Switzerland where 45% of the patients arriving at the hospital within 4 h after symptom onset receive thrombolysis. Therefore, reducing prehospital delays will increase thrombolysis rate and facilitate enrolment of patients in clinical trials in order to improve the poor outcome of CRAO.

## Data Availability Statement

The raw data supporting the conclusions of this article will be made available by the authors, without undue reservation.

## Author Contributions

EA, FB, JV, VS, and GK contributed to the conception and design of the study. EA, FB, and GK performed the statistical analysis. EA wrote the first draft of the manuscript. GK designed the research project and revised the manuscript. All authors contributed to the data collection of the study, commented on previous versions of the manuscript, read, and approved the final version of the manuscript.

## Conflict of Interest

The authors declare that the research was conducted in the absence of any commercial or financial relationships that could be construed as a potential conflict of interest.

## Publisher's Note

All claims expressed in this article are solely those of the authors and do not necessarily represent those of their affiliated organizations, or those of the publisher, the editors and the reviewers. Any product that may be evaluated in this article, or claim that may be made by its manufacturer, is not guaranteed or endorsed by the publisher.

## Abbreviations

CRAO: central retinal artery occlusion
SC/SU: Stroke center/Stroke unit
SSR: Swiss stroke registry
GP: General practitioner
DOAC: direct oral anticoagulants
TIA: transient ischemic attack
IVT: intravenous thrombolysis
IAT: intra-arterial thrombolysis.
