# Supplementary material for: Central Retinal Artery Occlusion: Current Practice, Awareness and Prehospital Delays in Switzerland
Source: Front Neurol. 2022 May 23;13:888456. doi: 10.3389/fneur.2022.888456 (PMC9167925; doi:10.3389/fneur.2022.888456)
Supplement: Supplementary file 1 [file Data_Sheet_1.pdf]

SUPPLEMENTAL MATERIAL

Table of contents:

1. Expanded methods - Questionnaires
   1.1. Questionnaire for the population
   1.2. Questionnaire for the ophthalmologist
   1.3. Questionnaire for the general practitioners

2. Supplemental tables from the data from the Swiss Stroke Registry
   2.1. Supplemental table 1: Aetiology
   2.2. Supplemental table 2: Adverse events and modified Ranking Scale (mRS) at 3 months

3. Supplemental result of the survey
   3.1. Supplemental table 3: Result of the survey in the population
   3.2. Supplement table 4: Result of the survey in the Ophthalmologist
   3.3. Supplement table 5: Result of the survey in the general practitioners

4. Supplement list of collaborators from the Swiss Stroke Registry

1. Expanded methods - Supplement Questionnaire

1.1. Questionnaire for the population

1. Sex
    a. Male
    b. Female

2. Age

3. Years of education

4. Do you have a medical background?
    a. Yes
    b. No

5. Which of the following are possible symptoms of a disturbance in blood supply in the eye?
    a. Pain in the eye
    b. Unilateral, acute visual loss
    c. Bilateral, acute visual loss
    d. Blurred vision
    e. Visual loss and headache

6. Which of the following sentence is true in cases of visual loss?
    a. The visual loss is in most cases only transient
    b. A vessel occlusion can cause it
    c. There is no need for therapy
    d. It is a medical emergency

7. What would you do in case of a visual loss in one eye?
    a. Wait until symptoms recover spontaneously
    b. Make an appointment with my general practitioner
    c. Make an appointment with my ophthalmologist
    d. I would search for a doctor only in case of persistent symptoms
    e. Go to the next emergency room immediately

8. In your opinion, who should treat an acute visual loss?
    a. General practitioner
    b. Ophthalmologist
    c. The nearest hospital center
    d. The nearest hospital

9. Have you ever heard of an eye infarction?
    a. Yes
    b. No

10. If yes, where?
    a. News/magazine
    b. Television
    c. Internet
    d. Friends
    e. General practitioner

11. In your opinion, how fast should a visual loss be treated?
    a. It is an emergency (within 2 hours)
    b. Within 4 hours
    c. Within 24 hours
    d. Within 48 hours

e. Within a week

12. In case the symptoms recover within a few minutes spontaneously, would you still search for a doctor?
    a. Yes
    b. No

13. Has any of your family members or friend ever had a disturbance of blood supply in an eye?
    a. Yes
    b. No

14. Has any of your family members or friends ever had a stroke?
    a. Yes
    b. No

15. What would you do in case of stroke symptoms?
    a. Wail until symptoms recover spontaneously
    b. Make an appointment with my general practitioner
    c. I would search for a doctor only in case of persistent symptoms
    d. Go to the next emergency room immediately

16. Would you like to receive further information on this topic?
    a. Yes
    b. No

1.2. Questionnaire for the ophthalmologist

1. Sex
    a. Male
    b. Female

2. Age

3. Year of medical exam

4. What would you do with patients with a central retinal artery occlusion?
    a. Treat the patient as an emergency in my doctor´s office
    b. I would transfer the patient as an emergency to the next stroke center
    c. I would transfer the patient as an emergency to the next hospital
    d. I would arrange an etiological exploration

5. Which of the following options are effective treatments in central retinal artery occlusion? (acute treatment and secondary prevention)
    a. Early treatment with acidum acetylsalicylic
    b. Systemic thrombolysis
    c. Endovascular thrombolysis
    d. Mechanical treatment (for example, reducing the intraocular pressure, Bulbusmassage)

6. In your opinion, is a central retinal artery occlusion an emergency, which needs a fast exploration and treatment?
    a. Yes
    b. No

7. In which time window can the visual loss in case of central retinal artery occlusion be reversible?
    a. < 4 hours
    b. < 8 hours
    c. < 24 hours
    d. < 48 hours

8. In case the symptoms recover within a few minutes, does the patient still need further exploration?
    a. Yes
    b. No

9. Which percentage of the patients with a central retinal artery occlusion shows a spontaneous recovery of visual acuity?
    a. < 30 %
    b. 42 %
    c. 50 %
    d. 78 %
    e. > 90 %

10. Which one is the most common etiology of the central retinal artery occlusion?
    a. Atherosclerosis / Stenosis of the internal carotid artery
    b. Arteriitis temporalis
    c. Atrial fibrillation
    d. Dissection of the internal carotid artery

11. Would you like to receive further information on this topic?
    a. Yes
    b. No

1.3. Questionnaire for the general practitioner

1. Sex
    a. Male
    b. Female

2. Age

3. Year of medical exam

4. How common is the central retinal artery occlusion (incidence per year)?
    a. 0.1 per 100,000 people
    b. 1 per 100,000 people
    c. 10 per 100,000 people
    d. 20 per 100,000 people

5. In the presence of which symptoms would you suspect a central retinal artery occlusion?
    a. Pain in the eye
    b. Unilateral, acute visual loss
    c. Bilateral, acute visual loss
    d. Blurred vision
    e. Visual loss and headache

6. Which of the following are possible differential diagnosis of an acute, painless, unilateral visual loss?
    a. Retinal detachment
    b. Central retinal artery occlusion
    c. Arteriitis temporalis (Morbus Horton)
    d. Optic neuritis
    e. Ischaemic optic neuropathy

7. Do you have personal experience with the diagnosis and treatment of central retinal artery occlusion?
    a. Yes
    b. No

8. If you have any experience with patients with central retinal artery occlusion, how many patients do you see in your doctor´s office per year?
    a. > 5 patients per year
    b. > 1 patient per year
    c. < 1 patient per year

9. What would you do in case of suspected central retinal artery occlusion?
    a. I would make an appointment with the ophthalmologist for the patient
    b. I would arrange an emergency transfer for the patient to the ophthalmologist
    c. I would arrange an emergency transfer for the patient to the next emergency room in a stroke center
    d. I would arrange an emergency transfer for the patient to the next emergency room in a peripheral hospital
    e. I would make an appointment for the next day in my doctor´s office
    f. I would monitor the patient in my doctor's office until the symptoms reverse
    g. I would start a secondary prevention
    h. I would arrange an etiological exploration

10. Which basic diagnostic would you do in your doctor's office?
    a. Measure the blood pressure
    b. ECG
    c. Blood test (BSR, hemogramm, CRP)
    d. Blood test (blood sugar)
    e. Carotid ultrasound

11. Which of the following treatments option are, in your opinion, effective?
    a. Acid acetylsalicylic
    b. Systemic thrombolysis
    c. Endovascular thrombolysis
    d. Mechanical treatment (for example, reducing the intraocular pressure, Bulbusmassage)
    e. There is no need for treatment

12. In which time window can the visual loss in case of central retinal artery occlusion be reversible?
    a. < 4 hours
    b. < 8 hours
    c. < 24 hours
    d. < 48 hours

13. In your opinion, is a central retinal artery occlusion an emergency, which needs a fast exploration and treatment?
    a. Yes
    b. No

14. Which percentage of the patients with a central retinal artery occlusion shows a spontaneous recovery of visual acuity?
    a. < 30 %
    b. 42 %
    c. 50 %
    d. 78 %
    e. > 90 %

15. Which one is the most common etiology of the central retinal artery occlusion?
    a. Atherosclerosis / Stenosis of the internal carotid artery
    b. Arteritis temporalis
    c. Atrial fibrillation
    d. Dissection of the internal carotid artery

16. Would you like to receive further information on this topic?
    a. Yes
    b. No

2. Supplemental tables from the Data from the Swiss Stroke Registry

2.1. Supplemental Table 1: Aetiology

	CRAO (all)	CRAO (revascularised)	CRAO (standard of care)	CRAO revasc. vs standard of care *p*-value	Ischemic stroke	CRAO (all) vs stroke *p*-value
Large artery atherosclerosis - n° (%)	115 (30.5)	11 (25)	86 (32.3)	0.331*	4500 (14.9)	0.000*
Cardiac embolism - n° (%)	40 (10.6)	4 (9.1)	31 (11.7)	0.799*	8287 (27.4)	0.000*
Small vessel disease - n° (%)	19 (5.0)	1 (2.3)	11 (4.1)	1.000*	3500 (11.6)	0.000*
Cervical artery dissection - n° (%)	8 (2.1)	1 (2.3)	7 (2.6)	1.000*	648 (2.1)	0.982*
PFO - n° (%)	4 (1.1)	0	3 (1.1)	1.000*	599 (2.0)	0.203*
Other determined etiology - n° (%)	37 (9.8)	3 (6.8)	24 (9.0)	0.779*	1843 (6.1)	0.003*
More than one possible aetiology - n° (%)	22 (5.8)	1 (2.3)	17 (6.4)	0.486*	1797 (5.9)	0.937*
Unknown aetiology despite complete evaluation - n° (%)	73 (19.4)	14 (31.8)	44 (16.5)	0.016*	4849 (16.0)	0.078*
Unknown aetiology with incomplete evaluation - n° (%)	59 (15.6)	9 (20.5)	43 (16.2)	0.481*	3662 (12.1)	0.035*
Stroke or TIA mimic – nº (%)	0				607 (2.0)	0.006*

*Chi-Square Test, Fisher's exact test is used when there is at least one cell in the contingency table of the expected frequencies below 5.

2.2. Supplemental Table 2: Adverse events and modified Ranking Scale (mRS) at 3 months

**Adverse events - acute**	CRAO (all)	CRAO (revascularised)	CRAO (standard of care)	CRAO revasc. vs standard of care *p*-value	Ischemic stroke	CRAO (all) vs stroke *p*-value
Intracranial hemorrhage	1 (0.3)	1 (2.3)	0	0.138*	459 (1.5)	0.047*

Stroke	5 (1.3)	1 (2.3)	4 (1.5)	0.525*	607 (2.1)	0.311*
epileptic seizure	0				344 (2.0)	0.016*
Angioedema	0				29 (0.6)	1.000*
Death	0				1750 (5.8)	0.000*
**Adverse events and mRS - after 3 months**						
Intracranial hemorrhage	0				68 (0.3)	1.000*
Stroke	7 (2.3)	2 (5.9)	4 (1.9)	0.203*	597 (2.8)	0.626*
epileptic seizure	1 (0.4)				152 (1.1)	0.737*
Death	12 (4.0)				1543 (7.2)	
- Fatal stroke or intracranial haemorrhage	1 (8.3)	0	1	0.371*	513 (3.5)	0.032*
- Ohter vascular death	2 (16.7)		2		73 (4.8)	
- Non-vascular death	2 (16.7)		1		325 (21.6)	
- unknown	7 (58.3)		7		596 (39.5)	
mRS nach 90 days						
- 0	69 (22.9)	6 (17.6)	56 (27.1)	0.245*	6478 (30.2)	0.006*
- 1	137 (45.5)	17 (50.0)	83 (40.1)	0.277*	5301 (24.7)	0.000*
- 2	60 (19.9)	10 (29.4)	39 (18.8)	0.156*	3313 (15.5)	0.033*
- 3	19 (6.3)	0	15 (7.2)	0.138*	2528 (11.8)	0.003*
- 4	5 (1.7)	1 (2.9)	4 (1.9)	0.536*	1871 (8.7)	0.000*
- 5	0	0	0	-	415 (1.9)	0.015*
- 6	11 (3.7)	0	10 (4.8)	0.365*	1531 (7.1)	0.019*

*Chi-Square Test, Fisher's exact test is used when there is at least one cell in the contingency table of the expected frequencies below 5.

mRS: modified Ranking Scale

3. Supplemental Result of the survey

3.1. Supplement Table 3: Result of the survey in the population

Questions	Positive Answer
1. Sex (male) – nº (%)	148 (42.3)
2. Mean age – SD	44 (15.57)
3. Medical background – nº (%)	72 (20.6)
4. Possible symptoms in case of disturbance in blood supple in the eye – nº (%)	
4.1. Pain in the eye	147 (42.0)
4.2. Unilateral, acute visual loss	100 (28.6)
4.3. Bilateral, acute visual loss	70 (20.0)
4.4. Blurred vision	196 (56.0)
4.5. Visual loss and headache	147 (42.0)
5. Which of the following sentence is true in cases of visual loss? – nº (%)	
5.1. The visual loss is in most cases only transient	72 (20.6)
5.2. A vessel occlusion can cause it	176 (50.3)
5.3. There is no need for therapy	10 (2.9)
5.4. It is a medical emergency	221 (63.1)
6. What would you do in case of a visual loss in one eye? – nº (%)	
6.1. Wait until symptoms recover spontaneously	7 (2.0)
6.2. Make an appointment with my general practitioner	47 (13.4)
6.3. Make an appointment with my ophthalmologist	120 (34.3)
6.4. I would search for a doctor only in case of persistent symptoms	40 (11.4)
6.5. Go to the next emergency room immediately	194 (55.4)
7. In your opinion, who should treat an acute visual loss? – nº (%)	
7.1. General practitioner	13 (3.8)
7.2. Ophthalmologist	167 (49.0)
7.3. The nearest hospital center	45 (13.2)
7.4. The nearest hospital	116 (34.0)
8. Have you ever heard of an eye infarction? (yes) – nº (%)	136 (39.5)
9. If yes, where? – nº (%)	
9.1. News/magazine	50 (14.3)
9.2. Television	47 (13.4)
9.3. Internet	40 (11.4)
9.4. Friends	67 (19.1)
9.5. General practitioner	19 (5.4)
10. In your opinion, how fast should a visual loss be treated? – nº (%)	
10.1. It is an emergency (within 2 hours)	205 (60.8)
10.2. Within 4 hours	51 (15.1)
10.3. Within 24 hours	57 (16.9)
10.4. Within 48 hours	10 (3.0)
10.5. Within a week	14 (4.2)
11. In case the symptoms recover within a few minutes spontaneously, would you still search for a doctor? (Yes) – nº (%)	239 (69.7)
12. Has any of your family members or friend ever had a disturbance of blood supply in an eye? (yes) – nº (%)	33 (9.7)
13. Has any of your family members or friends ever had a stroke? (yes) – nº (%)	142 (42.5)
14. What would you do in case of stroke symptoms? – nº (%)	
14.1. Wail until symptoms recover spontaneously	1 (0.3)
14.2. Make an appointment with my general practitioner	30 (8.8)

14.3. I would search for a doctor only in case of persistent symptoms	7 (2.0)
14.4. Go to the next emergency room immediately	304 (88.9)

3.2. Supplemental Table 4: Result of the survey in the Ophthalmologist

Questions	Positive Answer
1. Sex (male) – nº (%)	37 (55.2)
2. Median age – SD yr	45.5 (10.7)
3. What would you do with patients with a CRAO? – nº (%)	
3.1. Treat the patient in my doctor office	11 (16.4)
3.2. I would transfer the patient as an emergency to the next stroke center	43 (64.2)
3.3. I would transfer the patient as an emergency to the next hospital	2 (3.0)
3.4. I would arrange etiological exploration	0
	11 (16.4)
4. Which of the following options are effective treatments in CRAO? (acute treatment and secondary prevention) – nº (%)	
4.1. Early treatment with acidum acetylsalicylic	32 (47.8)
4.2. Systemic thrombolysis	16 (23.9)
4.3. Endovascular thrombolysis	28 (41.8)
4.4. Mechanical treatment (for example, reducing the intraocular pressure, Bulbusmassage)	54 (80.6)
5. Is a CRAO an emergency, which needs a fast exploration and treatment? (Yes) – nº (%)	66 (98.5)
6. In which time window can the visual loss in case of CRAO be reversible? – nº (%)	
6.1. < 4 hours	51 (76.1)
6.2. < 8 hours	15 (22.4)
6.3. < 24 hours	0
6.4. < 48 hours	1 (1.5)
7. In case the symptoms recover within a few minutes, does the patient still need further exploration? (Yes) – nº (%)	67 (100)
8. Which percentage of the patients with a CRAO shows a spontaneous recovery of visual acuity? – nº (%)	
8.1. < 30 %	62 (92.5)
8.2. 42 %	3 (4.5)
8.3. 50 %	2 (3.0)
8.4. 78 %	0
8.5. > 90 %	0
9. Which one is the most common etiology of the CRAO? – nº (%)	
9.1. Atherosclerosis / Stenosis of the internal carotid artery	53 (79.1)
9.2. Arteritis temporalis	3 (4.5)
9.3. Atrial fibrillation	11 (16.4)
9.4. Dissection of the internal carotid artery	0

CRAO = central retinal artery occlusion

3.3. Supplemental Table 5: Result of the survey in the general practitioners

Questions	Positive Answer
1. Sex (male) – nº (%)	79 (77.5)
2. Median age – SD yr	54.5 (9.42)
3. How common is the CRAO (incidence per year)? – nº (%)	
3.1. 0.1 per 100,000 people	5 (4.9)
3.2. 1 per 100,000 people	46 (45.5)
3.3. 10 per 100,000 people	44 (43.6)
3.4. 20 per 100,000 people	6 (5.9)
4. In the presence of which symptoms would you suspect a CRAO? – nº (%)	
4.1. Pain in the eye	1 (1.0)
4.2. Unilateral, acute visual loss	90 (88.2)
4.3. Bilateral, acute visual loss	1 (1.0)
4.4. Blurred vision	4 (3.9)
4.5. Visual loss and headache	6 (5.9)
5. Which of the following are possible differential diagnosis of an acute, painless, unilateral visual loss? – nº (%)	
5.1. Retinal detachment	84 (82.4)
5.2. Central retinal artery occlusion	95 (93.1)
5.3. Arteritis temporalis (Morbus Horton)	51 (50.0)
5.4. Optic neuritis	63 (61.8)
5.5. Ischaemic optic neuropathy	72 (70.6)
6. Do you have personal experience with the diagnosis and treatment of CRAO? (yes) – nº (%)	30 (29.4)
7. If you have any experience with patients with CRAO, how many patients do you see in your doctor´s office per year?	
7.1. > 5 patients per year	1 (1.0)
7.2. > 1 patient per year	19 (18.6)
7.3. < 1 patient per year	58 (56.9)
8. What would you do in case of suspected CRAO? – nº (%)	
8.1. I would make an appointment with the ophthalmologist for the patient	2 (2.0)
8.2. I would arrange an emergency transfer for the patient to the ophthalmologist	34 (33.3)
8.3. I would arrange an emergency transfer for the patient to the next emergency room in a Stroke center	61 (59.8)
8.4. I would arrange an emergency transfer for the patient to the next emergency room in a peripheral hospital	1 (1.0)
8.5. I would make an appointment for the next day in my doctor´s office	0
8.6. I would monitor the patient in my doctor's office until the symptoms reverse	0
8.7. I would start a secondary prevention	1 (1.0)
8.8. I would arrange an etiological exploration	3 (2.9)
9. Which basic diagnostic would you do in your doctor's office? – nº (%)	
9.1. Measure the blood pressure	93 (91.2)
9.2. ECG	59 (57.8)
9.3. Blood test (BSR, hemogramm, CRP)	67 (65.7)
9.4. Blood test (blood sugar)	42 (41.2)
9.5. Carotid ultrasound	17 (16.7)
10. Which of the following treatments option are, in your opinion, effective? – nº (%)	52 (51.0)

10.1. Acid acetylsalicylic	38 (37.3)
10.2. Systemic thrombolysis	55 (53.9)
10.3. Endovascular thrombolysis	10 (9.8)
10.4. Mechanical treatment (for example, reducing the intraocular pressure, Bulbusmassage)	4 (3.9)
10.5. There is no need for treatment	
11. In which time window can the visual loss in case of CRAO be reversible? – nº (%)	
11.1. < 4 hours	40 (39.2)
11.2. < 8 hours	42 (41.2)
11.3. < 24 hours	16 (15.7)
11.4. < 48 hours	2 (2.0)
12. In your opinion, is a CRAO an emergency, which needs a fast exploration and treatment? (Yes) – nº (%)	99 (100)
13. Which percentage of the patients with a CRAO shows a spontaneous recovery of visual acuity?	
13.1. < 30 %	57 (55.9)
13.2. 42 %	19 (18.6)
13.3. 50 %	18 (17.6)
13.4. 78 %	3 (2,9)
13.5. > 90 %	1 (1.0)
14. Which one is the most common etiology of the CRAO?	
14.1. Atherosclerosis / Stenosis of the internal carotid artery	53 (52)
14.2. Arteriitis temporalis	10 (9.8)
14.3. Atrial fibrillation	35 (34.3)
14.4. Dissection of the internal carotid artery	0

CRAO = central retinal artery occlusion

4. Supplemental list of collaborators from the Swiss Stroke Registry

List of collaborators – Swiss Stroke Registry.

The following collaborators participated in the Swiss Stroke Registry. They have confirmed that their names can be listed in any manuscript (i.e. appendix, acknowledgement) arising from this registry.

Center	***Collaborators***
**Stroke Center (alphabetical order)**	
Cantonal Hospital Aarau (KSA)	Sandra Clarke<br>Philipp Gruber<br>Timo Kahles<br>Eileen Martin<br>Krassen Nedeltchev<br>Luca Remonda<br>Andreas Schweikert<br>Vedrana Zupa
University Hospital Basel (USB)	Valerian Altersberger<br>Kristin Blackham<br>Leo H Bonati<br>Alex Brehm<br>Gian Marco De Marchis<br>Tolga Dittrich<br>Stefan T Engelter<br>Amgad El Mekabaty<br>Urs Fisch<br>Joachim Fladt<br>Henrik Gensicke<br>Lisa Hert<br>Philippe Lyrer<br>Sabrina Manuzzi<br>Marina Maurer<br>Alexandros Polymeris<br>Marios Psychogios<br>Sebastian Thilemann<br>Christopher Traenka<br>Ioannes Tsogkas<br>Benjamin Wagner<br>Annaelle Zietz
Inselspital, University Hospital Bern	Marcel Arnold<br>Urs Fischer<br>Martina Goeldlin<br>Jan Gralla<br>Mirjam Heldner<br>Simon Jung<br>Johannes Kaesmacher<br>Basel Maamari<br>Thomas R Meinel<br>Pasquale Mordasini

	Madlaine Mueller<br>Hakan Sarikaya<br>David Seiffge<br>Bernhard Siepen<br>Jan Vynckier<br>Morin Beyeler
University Hospital Geneva (HUG)	Iman Boukrid<br>Emmanuel Carrera<br>Elisabeth Dirren<br>Jose Bernardo Escribano Paredes<br>Nicolae Sanda
University Hospital Lausanne (CHUV)	Ashraf Eskandari<br>Patrick Michel<br>Vasiliki Pantazou<br>Davide Strambo
Cantonal Hospital Lucerne (LUKS)	Manuel Bolognese<br>Alexander von Hessling
Neurocenter of Southern Switzerland, Lugano (EOC)	Giovanni Bianco<br>Carlo W Cereda<br>Jane Frangi<br>Valentina Sciré
Cantonal hospital of St. Gallen (KSSG)	Georg Kägi<br>Jochen Vehoff<br>Johannes Weber<br>Anna Müller
University Hospital Zuerich (USZ)	Philipp Baumgartner<br>Meret Branscheidt<br>Annina Dietrich<br>Christoph Globas<br>Martin Hänsel<br>Janne Hamann<br>Jeremia Held<br>Mira Katan<br>Zsolt Kulcsar<br>Andreas R Luft<br>Achim Mueller<br>Theodor Pipping<br>Levke Steiner<br>Susanne Wegener<br>Laura Westphal<br>Roni Widmer<br>Jannie van Duinen<br>Annina Dietrich
Hirslanden Zürich	Bettina Anders<br>Roland Backhaus<br>Filip Barinka<br>Asterios Paliantonis<br>Nils Peters<br>Shadi Taheri
**Stroke Units (alphabetical order)**	

Cantonal Hospital of Baden	Alexander Tarnutzer
Spitalzentrum Biel (SZB)	Stephan Salmen
Cantonal Hospital Graubuenden Chur	Sylvan Albert<br>Rolf Sturzenegger
Cantonal Hospital Fribourg	Ettore Accolla<br>Jean-Marie Annoni<br>Léopold Colin-Benoit<br>David Cuendet<br>Sandrine Foucras<br>Andrea Humm<br>Friedrich Medlin
Sarganserland Stroke Unit Hospital Grabs	Christian Berger<br>Florian Lindheimer<br>Stephan Köppel
Spital Limmattal	Guido Schwegler
Cantonal Hospital Münsterlingen	Ludwig Schelosky
Cantonal Hospital Neuchâtel	Gabriele Brodo<br>Maria Cordier<br>Vaiva Jurgutiene<br>Peter Kelemen<br>Philippe Olivier<br>Susanne Renaud
GHOL Nyon	Loraine Fisch<br>Julien Niederhauser<br>Guillermo Toledo Sotomayor
Hôpital du Valais Sion	Christophe Bonvin
Bürgerspital Solothurn	Michael Schaerer
Stadtspital Waid und Triemli	Marie-Luise Mono
Cantonal Hospital Winterthur	Biljana Rodic
